# A Novel Self-Cleaving Viroid-Like RNA Identified in RNA Preparations from a Citrus Tree Is Not Directly Associated with the Plant

**DOI:** 10.3390/v14102265

**Published:** 2022-10-15

**Authors:** Beatriz Navarro, Shuai Li, Andreas Gisel, Michela Chiumenti, Maria Minutolo, Daniela Alioto, Francesco Di Serio

**Affiliations:** 1 Institute for Sustainable Plant Protection (IPSP), National Research Council (CNR), Via Amendola 122/D, 70126 Bari, Italy; 2 Institute of Plant Protection, Jiangsu Academy of Agricultural Sciences, Nanjing 210014, China; 3 Istituto di Tecnologie Biomediche, Consiglio Nazionale delle Ricerche, 70126 Bari, Italy; 4 International Institute of Tropical Agriculture, Ibadan 200001, Nigeria; 5 Dipartimento di Agraria, Università degli Studi di Napoli Federico II, 80055 Portici, Italy

**Keywords:** circular RNAs, infectious non-coding RNAs, satellite RNAs, viroids, hammerhead ribozyme, self-cleavage, high-throughput sequencing

## Abstract

Viroid and viroid-like satellite RNAs are infectious, circular, non-protein coding RNAs reported in plants only so far. Some viroids (family *Avsunviroidae*) and viroid-like satellite RNAs share self-cleaving activity mediated by hammerhead ribozymes (HHRzs) endowed in both RNA polarity strands. Using a homology-independent method based on the search for conserved structural motifs of HHRzs in reads and contigs from high-throughput sequenced RNAseq libraries, we identified a novel small (550 nt) viroid-like RNA in a library from a *Citrus reticulata* tree. Such a viroid-like RNA contains a HHRz in both polarity strands. Northern blot hybridization assays showed that circular forms of both polarity strands of this RNA (tentatively named citrus transiently-associated hammerhead viroid-like RNA1 (CtaHVd-LR1)) exist, supporting its replication through a symmetric pathway of the rolling circle mechanism. CtaHVd-LR1 adopts a rod-like conformation and has the typical features of quasispecies. Its HHRzs were shown to be active during transcription and in the absence of any protein. CtaHVd-LR1 was not graft-transmissible, and after its first identification, it was not found again in the original citrus source when repeatedly searched in the following years, suggesting that it was actually not directly associated with the plant. Therefore, the possibility that this novel self-cleaving viroid-like RNA is actually associated with another organism (e.g., a fungus), in turn, transiently associated with citrus plants, is proposed.

## 1. Introduction

Viroids and viroid-like satellite RNAs (Vd-LsatRNAs) are small, infectious, non-protein-coding and circular RNAs reported in plants only so far [1,2,3,4]. They differ from each other at the biological level, with viroids replicating and infecting their hosts in the absence of any helper virus, and Vd-LsatRNAs relying on a helper virus for their infectivity. Other small, circular, non-coding RNAs containing hammerhead ribozymes, differing from viroids and other infectious viroid-like RNAs, have been reported in plants, such as (i) the retroviroid-like RNA known as carnation stunt-associated viroid-like RNA [5] that is, however, non-infectious and has a DNA counterpart integrated in the genome of a pararetrovirus [5,6] and of the host plant [7], and (ii) retrozymes, which are ribozyme-containing retrotransposons [8].

According to structural, biological and functional features, viroids are classified in the families *Pospiviroidae* and *Avsunviroidae*. Members of the family *Pospiviroidae* adopt a rod-like or quasirod-like conformation of minimal free energy, which contains a central conserved region (CCR) involved in their nuclear replication mediated by an asymmetric rolling circle mechanism [9]. This replication pathway contemplates the formation of circular RNAs in only one polarity strand [10]. Viroids of the family *Avsunviroidae* lack the CCR but contain in both polarity strands the structural elements needed to form hammerhead ribozymes (HHRzs) [11]. These ribozymes are inactive in the rod-like or most stable branched conformations adopted by the circular viroid RNAs, but adopt the active conformation during transcription, thus, providing the self-cleaving activity needed to complete the viroid replication through a symmetric rolling circle mechanism. The hallmark of this replication mechanism is the accumulation in vivo of circular RNAs of both polarity strands [12,13]. HHRzs have also been reported in Vd-LsatRNAs. However, while viroid RNAs contain one HHRz in either polarity strand, VL-satRNAs may also contain the HHRz in only one RNA polarity. The other polarity strand may lack self-cleaving activity or may contain another ribozyme named hairpin [2]. Both viroid and Vd-LsatRNA populations infecting a single host are composed of closely related sequence variants, thus, showing the typical features of quasispecies [14] previously reported for viruses [15]. Sequence variability observed in the populations of these infectious agents generally preserve the most stable conformation of the RNA and some critical structural motifs of major relevance for their infectivity [16].

Whether viroids or Vd-LsatRNAs also exist in organisms other than plants is not known. However, indirect evidence was provided for two small circular RNAs containing HHRz in each polarity strand (cscRNA1 and cscRNA2), which have been isolated from cherry leaves [17,18]. They are not able to replicate autonomously in cherry plants and have been proposed to be Vd-LsatRNAs of mycoviruses associated with a fungus (*Apiognomonia erythrostomam*) infecting cherry leaves [19,20,21,22,23,24]. Recently, two new Vd-LRNAs containing aHHRz in each polarity strand, named fig hammerhead viroid-like RNA 1 and 2 (FHVd-LR1 and FHVd-LR2), have been reported from fig trees grown in Hawaii, but their biological nature remains undetermined [25]. These findings suggest that Vd-LRNAs containing HHRzs may be more common than currently thought and that their hosts may include organisms other than plants.

In the last few years, several new viroids of the family *Pospiviroidae* have been identified in plants by high-throughput sequencing and the following search for de novo assembled contigs with sequence similarity to viroids previously annotated in databases [26,27,28,29,30]. However, this approach is unable to identify novel viroids and viroid-like RNAs not sharing significant identity with those already known and is expected to be less efficient in the identification of HHRz-containing viroids. In fact, members of the family *Avsunviroidae* share little-to-no sequence identity. Instead, relationships between viroids of this family are based on structural features, such as the quasirod-like or branched secondary structure, tertiary interactions (i.e., kissing-loops) and the type of hammerhead ribozymes embedded in both polarity strands of their circular RNAs [11]. Therefore, it is not surprising that the most recently reported members of the family *Avsunviroidae*, apple hammerhead viroid, and a potential new viroid of the same family, grapevine hammerhead viroid-like RNA, were identified using HTS-based methods that are sequence homology-independent and identify potential novel viroids and/or viroid-like RNAs by looking for de novo assembled contigs with terminal direct repeats, a hallmark of potential circular RNAs [31,32].

In an attempt to identify novel viroids of the family *Avsunviroidae* and other viroid-like RNAs, we focused our attention on HHRz, a specific hallmark of these infectious agents to develop a homology-independent method to search for HHRzs in reads or de novo-generated contigs from HTS libraries. By searching in the RNA preparations from a citrus tree grown in the field, a novel Vd-LRNA containing one HHRz in either polarity strand was identified. The molecular and biological features of such a novel circular RNA, tentatively named citrus transiently-associated hammerhead viroid-like RNA 1 (CtaHVd-LR1) are presented and discussed here.

## 2. Materials and Methods

### 2.1. Plant Material, RNA Isolation and High-Throughput Sequencing

In the frame of a study aimed to identify the potential causal agent of cristacortis, a virus-like disease of still unknown etiology, a *Citrus reticulata* plant grown in Southern Italy (isolate 14A) and showing the typical symptoms of this disease was analyzed. Very young green sprouts were collected in spring 2018 and stored at −80 °C. A sample of the collected material (5 g) was extracted with buffer-saturated phenol and the preparation was enriched in double-stranded (ds) RNAs partitioning the nucleic acids by chromatography on non-ionic cellulose CF-11 (Whatman, Maidstone, UK) with STE (50 mM Tris-HCl, pH 7.2, 100 mM NaCl, 1 mM ethylenediaminetetraacetic acid (EDTA)) containing 16% ethanol [33]. Following digestion with TURBO DNase (Ambion, Foster City, CA, USA) and removing ribosomal RNAs (rRNAs) by the Ribo-Zero Plant leaf kit (Illumina, San Diego, CA, USA), an RNA-seq cDNA library was generated using the ScriptSeq v2 RNA-Seq Library Preparation Kit (Epicentre) according to the manufacturer’s protocol in which an initial denaturation step (95 °C for 5 min) was added. The cDNA library was pair-end sequenced (2 × 150) on an Illumina NextSeq500 analyzer (Illumina, San Diego, CA, USA). Nucleic acid preparations enriched in highly structured RNAs were obtained with buffer-saturated phenol followed by CF-11 chromatography, as described above [34], using STE buffer containing 35% ethanol instead of 16%. DNA extracts were obtained with the DNeasy Plant mini kit (Qiagen, Hilden, Germany) from 100 mg of tissue following the manufacturer’s instructions.

### 2.2. Bioinformatics Analysis

Trimmed and high quality filtered reads from the sequenced RNAseq library were assembled de novo using SPAdes [35], and to identify potential infecting viruses, the resulting contigs were searched for similar sequences in the National Center for Technology Information (NCBI) databases by BlastN and BlastX. The same datasets were used for searching for RNAs potentially containing hammerhead ribozymes by using PatSearch [36] and applying a specific pattern syntax developed to find conserved nucleotides and secondary structure motifs conserved in these ribozymes. HTS reads were mapped to a reference sequence using Bowtie [37]. Multiple alignments of nucleotide sequences were performed using Clustal Omega [38]. The secondary structure of minimal free energy of RNAs was predicted by RNAfold software [39]. Pairwise identities were calculated by Clustal Omega multiple alignment [38]. Confidently predicted domains, motifs and features were searched for in SMART [40].

### 2.3. RT-PCR, Cloning and Sequencing

dsRNA enriched preparations were reverse transcribed using Superscript IV reverse transcriptase (Invitrogen, Thermo Fisher Scientific, Waltham, MA, USA) and random primers following the manufacturer’s instructions. PCR amplification of the cDNA was performed using 2 μL of the reverse transcription reaction, specific primers (Table S1) at a final concentration of 0.5 μM, 200 μM of dNTPs, 3% of DMSO, 1× Phusion HF buffer and 0.4 units of Phusion High-Fidelity DNA polymerase (Thermo Fisher Scientific, Waltham, MA, USA) in a final volume of 20 μL. The cycling conditions were initial denaturation at 98 °C for 30 s, followed by 33 cycles at 98 °C for 10 s, 63 °C for 15 s, 72 °C for 15 s and a final extension step at 72 °C for 7 min. Amplified cDNAs of the expected sizes were purified on agarose-gel and an adenine-residue overhang was added at their 5′ ends by GoTaq DNA polymerase (Promega, Madison, WI, USA), cloned into a pGEM-T Easy vector (Promega, Madison, WI, USA) and sequenced by Sanger Sequencing Custom Service (Macrogen, Amsterdam, the Netherlands).

### 2.4. Northern Blot Hybridization Assays

Northern blot hybridization assays were performed as reported previously [41]. Briefly, one μg of nucleic acid preparations enriched in highly structured RNAs were separated on 5% polyacrylamide gel electrophoresis (PAGE) under denaturing conditions (8 M urea and 1× TBE buffer (89 mM Tris, 89 mM boric acid, 2.5 mM EDTA, pH 8.3)). RNA fragments generated by in vitro transcription of a dimeric construct of (-) FHVd-LR [25] and an ssRNA ladder (New England Biolabs, Ipswich, MA, USA) were used as molecular markers. After ethidium bromide staining of the gel, the nucleic acids were electrotransferred to a nylon membrane (Hybond-N, Amersham, Little Chalfont, UK) in 0.5× TBE buffer and hybridized in DIG easy Hyb (Roche Applied Science, Germany) at 65 °C with DIG-labeled riboprobes complementary to each polarity strand of CtaHVd-LR1. Hybridization signals, revealed with an anti-DIG alkaline phosphatase conjugate (AB fragments) and the chemiluminescence substrate CDP-star (Roche Diagnostics GmbH, Mannheim, Germany) following the manufacturer’s instructions, were visualized and recorded with a ChemiDoc Touch Imaging system (Bio-Rad, Hercules, CA, USA). The DIG riboprobes were generated by in vitro transcription of the pGEM-T easy vector containing the full-length cDNA of the sequence variant CtaHVd-LR1_3 (accession number OP418121) linearized with the appropriated restriction enzymes using a Dig-RNA labeling mix and quantified through direct detection via spot test following the protocol provided by the manufacturer (Roche Diagnostics GmbH, Mannheim, Germany).

### 2.5. In Vitro Transcription, Self-Cleavage and 5′ Rapid Amplification of cDNA Ends (RACE) Analysis

Monomeric transcripts of both polarity strands were obtained by in vitro transcription of the pGemT-easy plasmid containing the full-length cDNA sequence of CtaHVd-LR1 (variant 3). The recombinant plasmid was linearized by digestion with *Spe*I or *Nco*I and transcribed with T7 (Thermo Fisher Scientific, Wattham, USA) or SP6 RNA polymerase (New England Biolabs) to obtain HVd-VL1 RNA transcripts of plus and minus polarity, respectively. After degradation of the DNA template with RQ DNase (Promega, Madison, Wisconsin, USA), the in vitro transcription reaction was analyzed in a 5% PAGE under denaturing conditions (see above). The full-length monomeric RNAs and the 3′ self-cleavage products generated during the in vitro transcription were eluted from the acrylamide gels by excising the band and grinding with one volume of water-saturated phenol and one volume of elution buffer (100 mM Tris-HCl pH 8.9, 1 mM EDTA, 0.5% SDS) [34]. The RNAs were recovered by ethanol precipitation and used for analysis of self-cleavage and the 5′ RACE, respectively.

For the analysis of RNA self-cleavage, the eluted RNAs were resuspended in 1 mM EDTA pH 6, boiled for 2 min, snap-cooled on ice and then incubated at 40 °C for 1 h in self-cleavage buffer (50 mM Tris⋅HCl, pH 8, 5 mM MgCl2, 0.5 mM EDTA). The self-cleavage reaction was analyzed in 5% PAGE under denaturing conditions as indicated above. The 5′ terminal sequence of the eluted 3′ self-cleavage RNA products was determined by 5′ RACE as described [25] using the oligonucleotides indicated in Table S1.

## 3. Results and Discussion

### 3.1. Identification of an RNA Containing HHRzs by In Silico Search

Viroids of the family *Avsunviroidae* and most viroid-like RNAs contain one HHRz in either RNA polarity strand. The active conformation of these catalytic motifs assume a conserved secondary structure, with typical loops and stems. By comparing all natural HHRzs known so far, we developed a specific pattern syntax (Figure S1) to look for sequences adopting a similar conformation in the reads and/or in the de novo assembled contigs from RNAseq libraries sequenced by HTS. Since most HHRzs consist of approximately 60 nucleotides, it is expected that some of the 150 nt-long reads generated by HTS may contain the full-length HHRz sequence of an infecting viroid and or viroid-like RNA. When the search was performed using the reads sequenced from an RNAseq library generated in 2018 from young sprouts of the *C. reticulata* tree isolate 14A, more than 600 redundant reads containing the signatures of HHRzs were found. Most of these reads corresponded to two non-redundant reads that were mapped to a single contig of 651 nt (NODE_11827_length_651_cov_57.163636), which was composed of a sequence fragment of 550 nt followed by a partial direct repeat of 101 nt (Figure S2). As mentioned before, the presence of direct repeats in a contig is generally considered as a possible indication that it could correspond to a multimeric or circular RNA in vivo. In this specific case, the presence of a HHRz in each polarity strand of the same RNA further supported its possible circularity because such a situation is a typical hallmark of viroids of the family *Avsunviroidae* [11] and of most Vd-LsatRNAs and Vd-LRNAs reported so far [1,2]. However, pairwise identity with these viroids ranged from 37.7 to 55.9% and no significant identity with sequences in GenBank was found by BlastN searches.

The identified RNA contained one ORF encoding a potential polypeptide of approximately 100 amino acids (aa) in the plus polarity strand that, however, does not show any significant similarity with proteins in GenBank and does not contain any confidently predicted domain, motif and feature according to a search in SMART [40]. These findings supported the working hypothesis that the monomeric fragment could be a novel viroid-like RNA that, also considering its biological features (see below), has been tentatively named citrus transiently-associated hammerhead viroid-like RNA 1 (CtaHVd-LR1). Blast searches also found contigs with sequences almost identical (99%) to the genomic RNAs of citrus virus A (CiVA), a coguvirus previously reported in citrus [42], and contigs sharing high sequence identity with several mycoviruses (i.e., partitiviruses, chrysoviruses and totiviruses) that were likely only indirectly associated with the citrus isolate 14A, being infectious agents of fungi or other organisms potentially hosted by the field tree.

### 3.2. Circular Forms of Both Polarity Strands of CtaHVd-LR1 Do Exist

**Figure 1:**
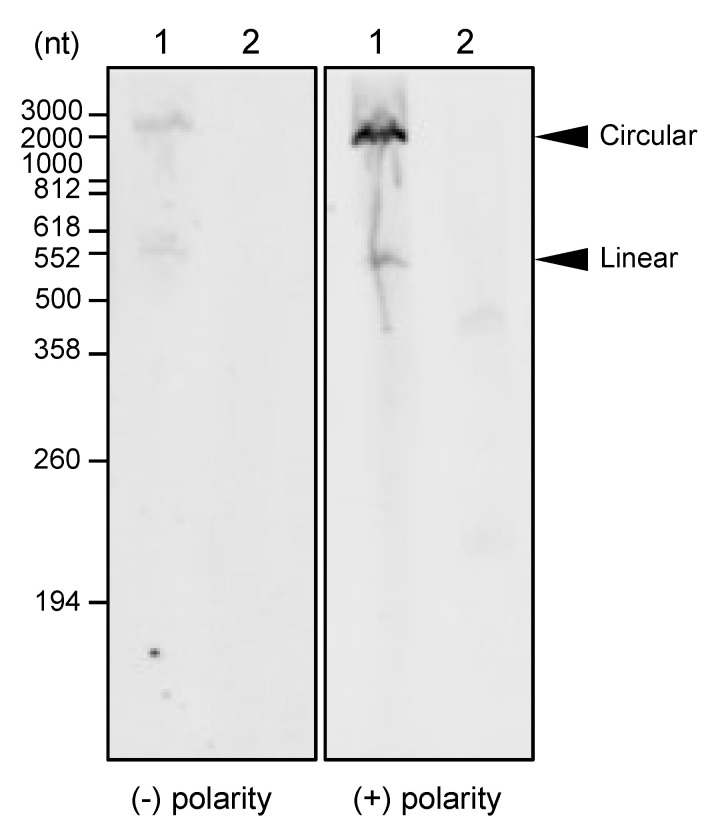
Detection of circular and linear forms of both polarity strands of CtaHVd-LR1 by northern blot hybridization assays. Lane 1, RNA preparation from citrus isolate 14A tested positive to CtaHVd-LR1 by HTS and RT-PCR; lane 2, RNA preparation from a sample of citrus isolate 14O tested negative to CtaHVd-LR1 by RT-PCR (negative control); size of RNA markers are indicated on the left. Identical aliquots of the same RNA preparations were loaded in parallel in the same gel; after nucleic acid transfer, the membrane was cut vertically, and each half membrane was hybridized with equalized DIG-probes to detect the (+) and the (−) polarity strand of CtaHVd-LR. Hybridization signals from both membranes were recorded simultaneously. The positions of circular and linear forms of CtaHVd-LR1 are indicated on the right. The (+) polarity was assigned, by convention, to the CtaHVd-LR1 polarity strand that accumulates at a higher level in vivo and generated a stronger hybridization signal (left panel).

Circular RNAs can be separated from the respective linear forms by polyacrylamide gel electrophoresis (PAGE) under denaturing conditions that determine a delayed migration of circular RNAs with respect to the linear ones [43]. To ascertain whether CtaHVd-LR1 exist as a circular RNA, we performed northern blot assays using probes specific for each polarity strand. Two major bands were detected in the nucleic acid preparations enriched in highly structured RNAs extracted from citrus sprouts collected in 2018 using specific probes for either polarity strand of this RNA (Figure 1), with the upper band identified as the circular form and the faster migrating band as the monomeric CtaHVd-LR1 linear form with an expected size of 550 nt. By preliminary northern blot assays using in vitro CtaHVd-LR1 RNA transcripts, we excluded cross-hybridization between CtaHVd-LR1 strands of the same polarity under the experimental conditions adopted in this study (Figure S3). Therefore, since equalized probes and RNA preparations were used, the more intense hybridization signal generated by one polarity strand also indicated that this is the polarity accumulating at higher levels in vivo, to which, as previously conducted for viroids and other viroid-like RNAs [1,2], the (+) polarity was assigned. These findings support the view that CtaHVd-LR1 very likely replicates through the symmetric pathway of the rolling circle replication mechanism previously proposed for viroids of family *Avsunviroidae* [44] and other viroid-like RNAs containing self-cleaving ribozymes in both polarity strands [2]. Indeed, the detection of circular RNAs of both polarity strands, together with the presence of self-cleaving ribozymes in both polarity strands, are generally considered the hallmark of this replication mode [1].

### 3.3. The Sequence Variability Detected in CtaHVd-LR1 Populations Preserves a Rod-Like Secondary Structure of Minimal Free Energy

**Figure 2:**
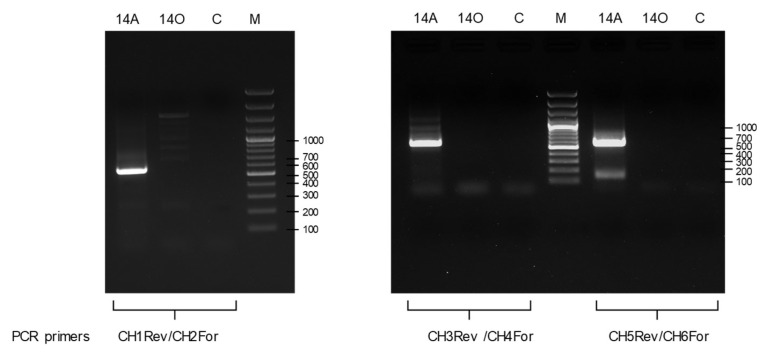
Amplification assays using RNA preparations from CtaHVd-LR1-positive (isolate 14A in 2018) and -negative (14O) samples and primer pairs CH1Rev/CH2For (**left**). CH3Rev/CH4F (**middle**) or CH5Rev/CH6F (**right**); C, negative control in which ddH_2_O instead of cDNA was added to the amplification mix; M, molecular marker is a 100 bp DNA ladder.

**Figure 3:**
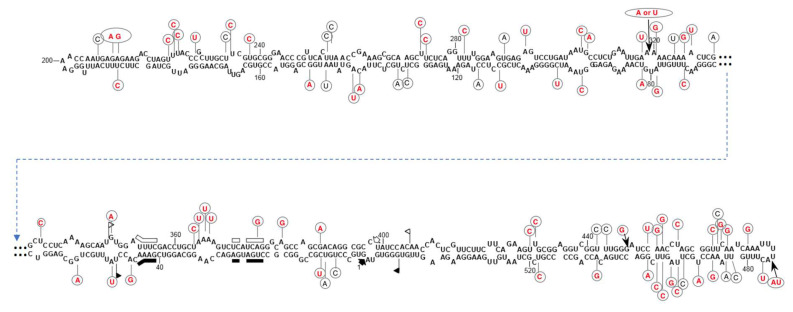
Primary and predicted secondary structures of lowest free energy of CtaHVd-LR1 reference variant (accession number OP418121). Regions involved in the formation of (+) and (−) HHRz structures and the respective self-cleavage sites are indicated by flags and thick arrows, respectively, with bars demarking the nucleotides conserved in most natural hammerhead structures. Filled and open symbols refer to (+) and (−) polarity, respectively. Polymorphic positions identified in the multiple alignment with the other cloned variants are indicated by circles containing the mutated nucleotide. Nucleotide changes preserving the proposed rod-like secondary structure are in red, with insertions marked by thin arrows.

The existence of CtaHVd-LR1 in the original RNA preparations used for generating the RNAseq library was initially confirmed by RT-PCR using specific primers designed in the contig (Table S1, data not shown). New total RNA preparations, extracted using different aliquots of the original sample of young sprouts (collected in 2018 and stored at −80 °C), were also tested by RT-PCR using three different specific primer pairs, adjacent and of opposite polarity, that were designed to amplify full-length cDNAs of the circular CtaHVd-LR1 (Table S1). With all primer pairs, amplicons of the expected size were obtained (Figure 2), confirming the identification of circular CtaHVd-LR1 RNAs with an alternative method. Cloning of the amplification products and sequencing of a total of 30 clones (Figure S4) showed they corresponded to sequence variants of CtaHVd-LR1 identified in silico and confirmed that their size is from 550 to 551 nt, with a G + C content of 49.5%, which is a value typical of other VL-RNAs. Since the amplicons were generated using three different primer pairs in independent reactions, we had the opportunity of investigating the variability in the regions targeted by each primer pair by sequencing the amplicons generated with the other pairs of primers, finally assessing the sequence variability of the complete CtaHVd-LR1 RNA (Figure S4). The sequenced CtaHVd-LR1 variants were between 99.1 and 99.7% identical to each other and their multiple alignment allowed the identification of sequence variability at several positions in the CtaHVd-LR1, with approximately 90 polymorphic positions identified in the multiple alignment (Figure S4). The consensus sequence of the multiple alignment shared the highest identity with the variant CtaHVd-LR1_3 that was, therefore, considered as the reference CtaHVd-LR1 variant (accession number OP418121). The secondary structure of minimum free energy of this variant corresponded to a rod-like conformation in which 73.8% of the residues were paired (Figure 3).

In the proposed rod-like structure, the central region contains the HHRzs of the two polarity strands that were located one in front of the other. Interestingly, most polymorphic positions did not modify the proposed rod-like secondary structure because they were co-variations, conversions of canonical into wobble base-pairs or were located into loops (Figure 3). It is worthy of note that several polymorphic positions map to the central hammerhead region (see below). Altogether, data on the sequence variability of CtaHVd-LR1 populations preserving the predicted rod-like secondary structure suggest a major role of this structural feature for its survival.

### 3.4. HHRzs Embedded in CtaHVd-LR1 Are Active during Transcription and in the Absence of Proteins

**Figure 4:**
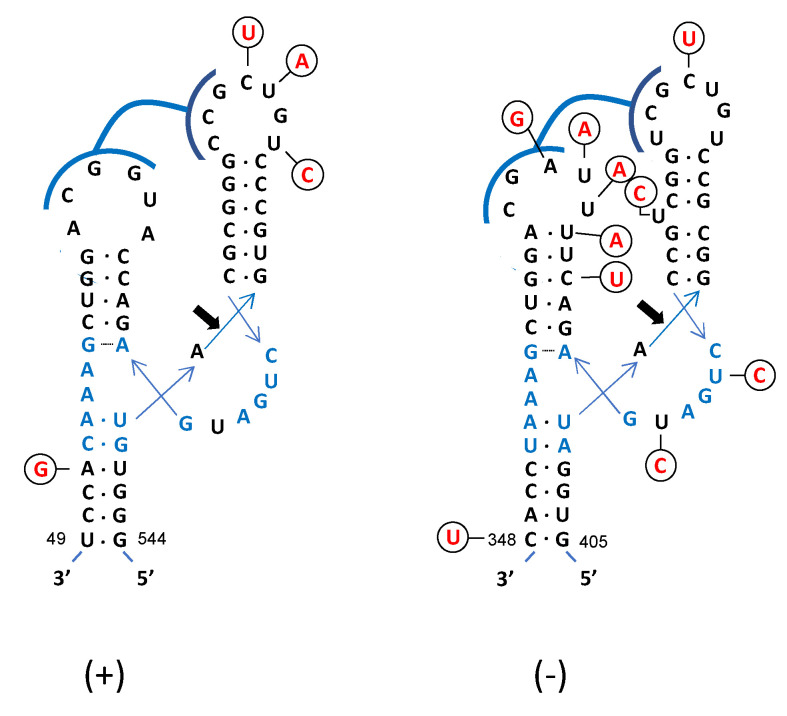
Primary and secondary structure of hammerhead ribozymes (HHRzs) of (+) and (−) CtaHVd-LR1. The nucleotides of the catalytic core conserved in most natural hammerhead structures are in blue. The cleavage site of each ribozyme is indicated by an arrow. Nucleotides in the loops of both HHRzs potentially involved in tertiary interactions are marked by blue lines. Nucleotides in the (+) and (−) polarity are numerated considering their positions in the variant CtaHVd-LR1_3. Mutations observed in other variants are indicated in circles, with the compensatory mutations and those not modifying the proposed secondary structure of the HHRzs reported in red.

The CtaHVd-LR1 hammerhead ribozymes have a central catalytic core surrounded by three hairpins, two of which (hairpin I and II) are closed by small loops (Figure 4). The self-cleavage site is preceded by atypical GUA and AUA trinucleotides in the (+) and (−) polarity strands, respectively. Such a combination of trinucleotides preceding the self-cleavage site was previously only reported in the (+) and (−) HHRzs of cscRNA1 and cscRNA2, two viroid-like RNAs reported to most likely be satellite RNAs of a mycovirus [24]. The GUA trinucleotide was also present at the same positions of the (+) HHRz of FHVd-LR [25] and in the (−) HHRz of the Vd-LsatRNAs of velvet tobacco mottle virus and lucerne transient streak virus [45,46]. The AUA was previously reported in the (+)-strand RNA of the satellite of barley yellow dwarf virus [47] and two novel viroid-like RNAs likely associated with carrot red leaf virus [48]. The sequence variability observed in the HHRzs always preserved the predicted active ribozyme structure because most changes were mapped at the hammerhead loops, preserving the potential tertiary interactions between some nucleotides, or did not modify base pairing of the stems (Figure 4). In four of the sequenced variants, one change was found in the catalytic core of the (−) HHRz where the U at position 377 was replaced by a C, thus, modifying the conserved CUGA motif into a CCGA motif (Figure 4). The same mutation has been previously reported in one variant of peach latent mosaic viroid (PLMVd), and it has been shown that it did not affect the self-cleaving capability of the mutated HHRz [49].

**Figure 5:**
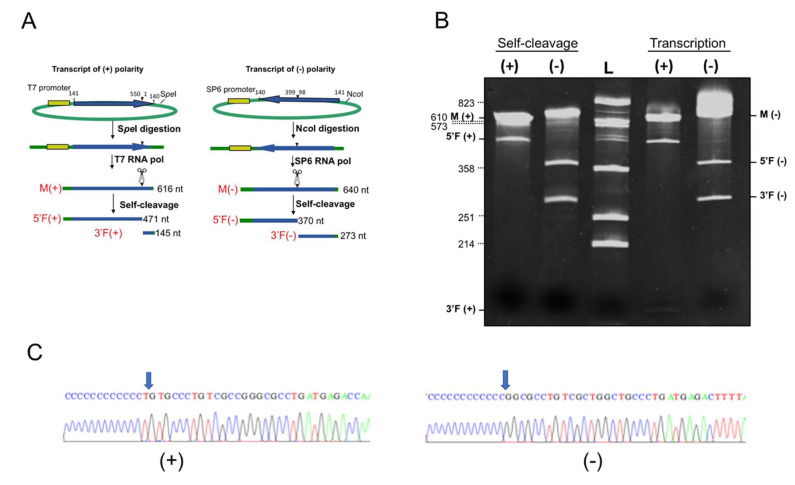
(**A**) Schematic representation of the plasmids and the RNA products generated by in vitro transcription. Plasmids containing the monomeric cDNA sequence of CtaHVd-LR1_3 in opposite orientations were linearized and transcribed to produce monomeric RNAs (M) of (+) and (−) polarity strands and the respective 5′ (5′F) and 3′ (3′F) fragments (left and right, respectively) derived from the HHRz self-cleaving activity. Plasmid sequences are depicted in green, while in yellow and blue are the polymerase promoter and CtaHVd-LR1 sequences, respectively. The scissors and arrowhead mark the position of the self-cleavage sites. The expected size of each fragment is reported on the right. (**B**) Analysis by 5% PAGE under denaturing conditions of the in vitro transcription (left) and self-cleavage reaction in the absence of any protein (right) of the plasmids containing (+) or (−) monomeric CtaHVd-LR1_3 cDNA. L, RNA ladder with sizes indicated on the left; see panel A for the abbreviations M, 3′F and 5′F. (**C**) Determination of self-cleavage site by 5′ RACE of 3′F fragment. Sequencing electropherograms of 5′ RACE products of the (+) and (−) 3′F fragments are shown on the left and right, respectively, with the 5′ terminal nucleotide (G) indicated by the arrow. The extra T observed at the terminal end of the (+) 3′F fragment cDNA (left) correspond to a non-template nucleotide that has likely been added by the reverse transcriptase during the cDNA synthesis [50].

The preservation of the hammerhead active structure, regardless of the sequence variability, is indirect evidence that these motifs play a key biological role. We provided experimental evidence that the HHRzs of CtaHVd-LR1 of both polarity strands are active during transcription, generating RNA fragments with sizes consistent with those expected considering the activity of HHRzs in the nascent RNA transcript (Figure 5). Moreover, when monomeric RNA transcripts were eluted from the gel, denatured and slowly cooled in a self-cleavage buffer containing 5 mM MgCl_2_, most of the transcript self-cleaved, generating the expected RNA fragments (Figure 5). Therefore, the HHRzs of both polarity strands of CtaHVd-LR1 are also active in the absence of any protein, confirming the ribozymatic nature of these catalytic motifs.

5′ RACE experiments followed by cloning and sequencing of the amplification products showed that the 3′ fragment resulting from the HHRz activity in the (−) polarity strand had the expected nucleotide at the 5′ terminus. In the (+) polarity strand, an extra nucleotide was observed at the 5′ terminus of the 3′ fragment generated by the (+) HHRz (Figure 5). However, this is a non-template nucleotide added to the 3′ end of the synthesized cDNA by the well-documented terminal nucleotidyl transferase activity of reverse transcriptases [50]. Altogether these data confirmed the predicted self-cleavage sites for the HHRz of both polarity strands of CtaHVd-LR1, thus, supporting their major role in the replication of this RNA.

### 3.5. CtaHVd-LR1 Is Not Associated with a DNA Counterpart

Since HHRzs have also been reported in circular RNAs that have a DNA counterpart integrated in the host genome (i.e., retrozymes or retroviroids) [5,6,7,8], we investigated whether this is the case for CtaHVd-LR1. DNA and dsRNAs were extracted from the original young sprouts of isolate 14A (collected in 2018 and stored at −80 °C) and tested by PCR and RT-PCR, respectively, using the primer pair CH2 For/CH5 Rev (Table S1). The expected amplicons were detected only by RT-PCR. In contrast, no amplicon was generated by PCR using the DNA preparation, thus, excluding the presence of a CtaHVd-LR1 DNA counterpart (Figure S5).

### 3.6. Assessment of the Biological Nature of CtaHVd-LR1

While RT-PCR assays performed using RNA preparations from the original sample of sprouts collected in 2018 from the isolate 14A were always positive, regardless of the primer pair (Table S1) used, all the attempts to detect CtaHVd-LR1 with the same primers in samples collected in 2019, 2020 and 2021 (early spring) from the same isolate always failed. The RT-PCR assays repeated in all seasons during 2020, including several times in early spring in 2021, always tested negative to CtaHVd-LR1. To exclude that the viroid-like RNA was not detected due to its low concentration, a new RNA library was prepared from sprouts of the isolate 14A collected in 2021 and sequenced by HTS. Contigs of CiVA were easily identified by de novo assembling of the sequenced reads followed by BlastN and BlastX searches. Moreover, when the sequenced reads were mapped on the CiVA reference genomic RNAs by Bowtie, a full coverage of the virus genome (average coverage depth 52 and 121 for RNA1 and RNA2, respectively) was observed, showing that the HTS was deep. Although 2 contigs related to partitiviruses were identified, no contig sharing significant sequence identity with chrysoviruses and totiviruses were identified in this second RNAseq library, suggesting that the mycoviruses detected in 2018 were not associated with the isolate 14A in 2021, likely because their primary host(s) was not infecting the plant when the samples were collected. Importantly, no contig sharing sequence identity with CtaHVd-LR1 was found, and no read was retrieved when the mapping by Bowtie was performed using CtaHVd-LR1 as a reference sequence, confirming the absence of this viroid-like RNA in the isolate 14A in the sample collected in 2021 and tested by HTS.

In line with the results reported above, the attempts of transmitting CtaHVd-LR1 to two grapefruit and two sour orange seedlings by grafting bark tissues from stems collected from the isolate 14A in early spring 2018 also failed, as confirmed by the negative results obtained when samples from the inoculated seedlings were tested for the next four years by RT-PCR. In contrast, in all the inoculated seedlings, CiVA was detected by RT-PCR already six months post-inoculation and during the following years, confirming that this virus was effectively graft-transmitted to the indicator plants and that the infection was stably maintained over time.

Altogether, these data support the view that CtaHVd-LR1 was only transiently associated with the citrus isolate 14A in 2018 and, possibly, that this circular RNA is not able to infect this host. Instead, considering the results of this study, the alternative possibility that CtaHVd-LR1 was infecting another organism transiently associated to the isolate 14A appears more feasible.

## 4. Conclusions

The identification of CtaHVd-LR1, by looking for conserved motifs of HHRzs, showed that homology-independent methods based on the identification of structural elements may be an effective method to find new viroid-like RNAs. With respect to the previously reported homology-independent methods, the one used in this study does not rely on the effective de novo assembly of the full genome plus terminal repeats of the viroid or viroid-like RNA for its identification. Indeed, when a HHRz is identified in a read that is not assembled in a contig with terminal repeats, adjacent primers of opposite polarities can be designed in the read of interest to check, by RT-PCR, whether the HHRz is part of a potential circular RNA that can be further characterized by sequencing full-length cDNAs and by northern blot hybridization assays.

By showing that CtaHVd-LR1 is a circular RNA endowed of an active HHRz in either polarity strand, we provided solid evidence that it is indeed a novel viroid-like RNA, confirming the reliability of the searching method. The detection of circular RNA forms of both polarity strands strongly support that this RNA replicates through the symmetric pathway of a rolling circle mechanism previously reported for several viroids and viroid-like RNAs [1,2]. The biological nature of CtaHVd-LR1 remains unknown. In this respect, CtaHVd-LR1 resembles cscRNAs and FHVd-LRs reported previously from cherry and fig trees, respectively [17,25]. As in the case of CtaHVd-LR1, these RNAs were also not graft-transmissible and were associated with mycoviruses [24,25]. In the case of cscRNAs, the mycoviruses were characterized as new species in the genera *Chrysovirus*, *Partitivirus* and *Totivirus* [19,20,21,22,23]. Interestingly, CtaHVd-LR1 was also identified in the isolate 14A together with contigs with the highest sequence identity to viruses of the genera *Chrysovirus*, *Partitivirus* and *Totivirus*. The fact that CtaHVd-LR1 was not found in the same isolate in the following years strongly supports the conclusion that it was only transiently, and possibly indirectly, associated with citrus plants. In this respect, the possibility that it could be an infectious agent of an organism other than citrus is quite likely. In conclusion, our study highlights the high sensitivity of HTS approaches associated with homology-independent bioinformatics tools to identify novel viroid-like RNAs. At the same time, we showed that the finding of a novel viroid-like RNA in a plant RNAseq library must not be considered as evidence of a direct association of the former with the latter. As also previously reported for plant viruses [51,52], the possibility that the viroid-like RNAs are only transiently associated with the plant, because actually infecting another organism must be considered, thus, highlights the need for implementing bioinformatics with biological data to clarify the nature of novel viroid-like RNAs.

## Data Availability

Full-length sequence variants of hammerhead viroid-like RNA 1 are available in GenBank (accession numbers OP418120-OP418149).

## Supplementary Materials

The following supporting information can be downloaded at: https://www.mdpi.com/article/10.3390/v14102265/s1, Table S1: Primers used in this study; Figure S1: Pattern syntax used to search for hammerhead ribozyme structure in citrus RNA seq library; Figure S2: Contig containing the sequences conserved in most natural hammerhead ribozymes in each polarity strand and a terminal direct repeat; Figure S3: CtaHVd-LR1 (+) or (−) transcript RNAs detected by northern-blot hybridization; Figure S4: Multiple-sequence alignment of full-length cDNAs of citrus transiently associated hammerhead viroid-like RNA 1 variants; Figure S5: PCR and RT-PCR assays using DNA preparations from isolate 14A.
