# Supplementary material for: A Novel Self-Cleaving Viroid-Like RNA Identified in RNA Preparations from a Citrus Tree Is Not Directly Associated with the Plant
Source: Viruses. 2022 Oct 15;14(10):2265. doi: 10.3390/v14102265 (PMC9608096; doi:10.3390/v14102265)
Supplement: Supplementary file 1 [file viruses-14-02265-s001.zip › viruses-1962659-supplementary.pdf]

Article

A Novel Self-Cleaving Viroid-Like RNA Identified in RNA Preparations from a Citrus Tree Is Not Directly Associated with the Plant

Beatriz Navarro [1], Shuai Li [1,2], Andreas Gisel [3,4], Michela Chiumenti [1], Maria Minutolo [5], Daniela Alioto [5] and Francesco Di Serio [1,*]

1 Institute for Sustainable Plant Protection (IPSP), CNR, Via Amendola 122/D, 70126 Bari, Italy;
2 Institute of Plant Protection, Jiangsu Academy of Agricultural Sciences, Nanjing, 210014, China;
3 Istituto di Tecnologie Biomediche, Consiglio Nazionale delle Ricerche, 70126 Bari, Italy;
4 International Institute of Tropical Agriculture, Ibadan 200001, Nigeria;
5 Dipartimento di Agraria, Università degli Studi di Napoli Federico II, 80055 Portici (NA), Italy.
* Correspondence: francesco.diserio@ipsp.cnr.it

Supplementary material

Table S1. Primers used in this study

Figure S1. Pattern syntax used to search for hammerhead ribozyme structure in citrus RNA seq library

Figure S2. Contig containing the sequences conserved in most natural hammerhead ribozymes in each polarity strand and a terminal direct repeat.

Figure S3. CtaHVd-LR1 (+) or (−) transcript RNAs detected by Northern-blot hybridization with equalized full-length digoxigenin-labeled riboprobes.

Figure S4. Multiple-sequence alignment of full-length cDNAs of hammerhead viroid-like RNA 1 variants.

Figure S5. PCR and RT-PCR assays using DNA and RNA preparations from isolate 14A, respectively.

Table S1. Primers used in this study

Name	Sequence (5' to 3')	position*	Used for
CH1 Rev	GTAAGAGCAGAGCCCTCATTTCTA	140-117	PCR of full-lengh CtaHVd-LR1 cDNA (coupled with CH2 For) and 5' RACE of the 3' fragment generated by the (+) HHRz self-cleavage
CH2 For	AGTATAATGGCGAGGTACCGTG	141-163	PCR of full-lengh CtaHVd-LR1 cDNA (coupled with CH1 Rev) and 5' RACE of the 3' fragment generated by the (-) HHRz self-cleavage
CH3 Rev	CCTTGGTCTCATCAGGCGC	34-17	PCR of full-lengh CtaHVd-LR1 cDNA (coupled with CH4 For)
CH4 For	CAGGTCGAAACACCTATTTGCT	35-56	PCR of full-lengh CtaHVd-LR1 cDNA (coupled with CH3 Rev)
CH5 Rev	AAACCGACCTCCGCAACTTCTG	444-423	PCR of full-lengh CtaHVd-LR1 cDNA (coupled with CH6 For)
CH6 For	GGGATCCAACCTAGCGGTTAAAT	445-468	PCR of full-lengh CtaHVd-LR1 cDNA (coupled with CH5 Rev)

* referred to variant CtaHVd-LR1_3

```
--------------------------------------------------------------------------------
 * COMMAND
--------------------------------------------------------------------------------
  //=============================================================================
  // COMMAND Hammer Head
  //=============================================================================

  //-----------------
  // Hammer Head
  //-----------------

  R110 = {AT, TA, GC, CG, TG, GT}

  L110 = 3...5 // Stem I

  L120 = RTM

  L130 = 3...7 // Stem II
  L131 = 3...15 // loop II

  L140 = CTGANGA
  L141 = 0...2

  L150 = G

  L160 = 3...20 // Stem III

  L170 = 3...50 // loop III

  L180 = C

  L190 = GAAAY

  //=============================================================================
  // PATTERN SYNTAX
  //=============================================================================


  //-----------------
  // Hammer Head
  //-----------------

  P1 = L110   L120   P2 = L130   L131   R110~P2  L141 L140  L141   L150   P3 = L160   L170 R110~P3   L180  L141   L190 R110~P1
```

Figure S1. Pattern syntax used to search for hammerhead ribozyme structure in citrus RNA seq library

Monomer (550 nt)

Direct repeat

>NODE_11827_length_651_cov_57.163636
TTGAAGGAAGAAGTTG**TGGGTGTAGTGCCCTGTCGCCGGGCGCCTGATGAGACCAAGGCA**
GGTCGAAACACCTATTTGCTGGCGAGGTCCGGGAACCTTGTTATGTCAAAAGAGAGGGTA
AATCGGGGAAACTCGCCTCCTAGAAATGAGGGCTCTGCTCTTACAGTATAATGGCGAGGT
ACCGTGCAGTTAGCAAGGGATTGCTAGCTTCTTTCATTTGGGAAACCAATGAAGGAAGAC
CTAGTTTACCCGCTTGCTTCGTGCGGGAACCCGTTCACTTAACCGAAAGCGCAAGCTTCT
CAGGTTTTGGAAGAGAGTGTCCTGATAACACCTCTGAATTGAAAAACAAGACTCGGCTCC
TCAAAAGCAA**TGTGGATTTCGACCTGCTAAAAGTCTCATCAGGGCAGCCAGCGACAGGCG**
CCTATCCACAACCACTCGTTCTTCTTCAGAAGTCGCGGAGGTCGGTTTGGGATCTAACCT
AGCGGTTAAATCAAATTTTACTTTGTAAAACCTGCTTAGTTCAGACCTGACACCAGCCCC
GTCGCTAATGTTGAAGGAAGAAGTTGTGGGTGTAGTGCCCTGTCGCCGGGCGCCTGATGA
GACCAAGGCAGGTCGAAACACCTATTTGCTGGCGAGGTCCGGGAACCTTGT

Figure S2. Contig containing the sequences conserved in most natural hammerhead ribozymes in each polarity strand (red and light-blue colors) and a terminal direct repeat (yellow background).

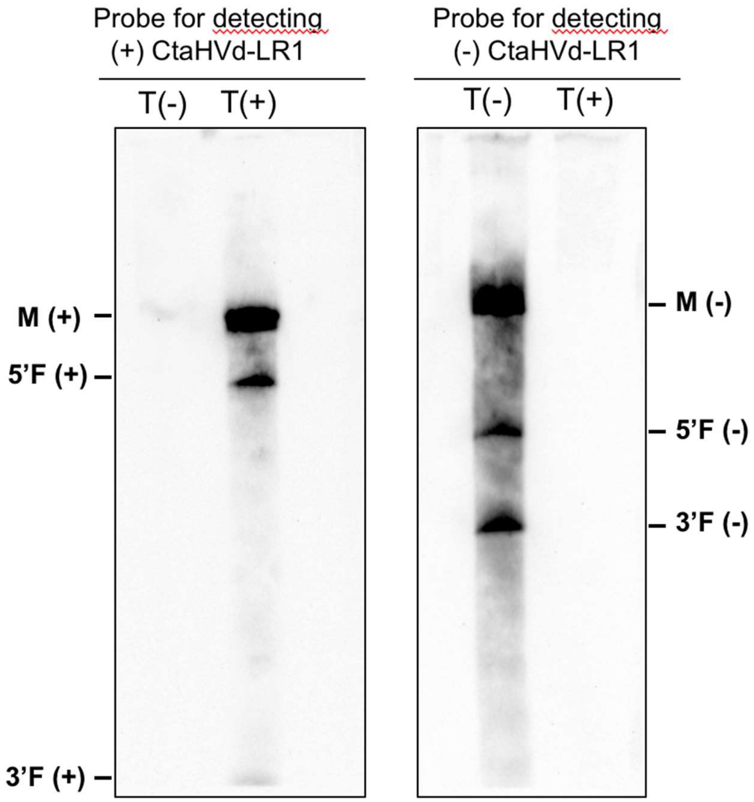

Figure S3. CtaHVd-LR1 (+) or (−) transcript RNAs detected by Northern-blot hybridization with equalized full-length digoxigenin-labeled riboprobes (left and right panel, respectively). Equal amounts of monomeric CtaHVd-LR1 (+) and (−) transcripts were loaded in lanes T(+) and T(-), respectively. The monomeric transcript (M) and the 5′F and 3′F fragments generated by the HHRzs are indicated.

```
                          Primer CH3 Rev     Primer CH4 For

CtaHVd-LR1_3        GTGCCCTGTCGCCGGGCGCCTGATGAGACCAAGGCAGGTCGAAACACCTATTTGCTGGCG   60
CtaHVd-LR1_36       ......C.....................................................   60
CtaHVd-LR1_22       ........................................................A...   60
CtaHVd-LR1_17       .........T..................................................   60
CtaHVd-LR1_23       ............................................................   60
CtaHVd-LR1_20       ........................................................A...   60
CtaHVd-LR1_24       ............................................................   60
CtaHVd-LR1_19       ............................................................   60
CtaHVd-LR1_18       ............................................................   60
CtaHVd-LR1_21       ............................................................   60
CtaHVd-LR1_11       ............................................................   60
CtaHVd-LR1_5        ............................................................   60
CtaHVd-LR1_10       ........................................................A...   60
CtaHVd-LR1_2        ........................................................A...   60
CtaHVd-LR1_33       ........A...................................................   60
CtaHVd-LR1_32       ............................................................   60
CtaHVd-LR1_31       ............................................................   60
CtaHVd-LR1_14       .............................................G..............   60
CtaHVd-LR1_9        ............................................................   60
CtaHVd-LR1_35       ............................................................   60
CtaHVd-LR1_12       .................................................T..........   60
CtaHVd-LR1_6        ............................................................   60
CtaHVd-LR1_28       ............................................................   60
CtaHVd-LR1_8        ............................................................   60
CtaCtaHVd-LR1_34    ............................................................   60
CtaHVd-LR1_29       ........................................................A...   60
CtaHVd-LR1_27       ............................................................   60
CtaHVd-LR1_16       ............................................................   60
CtaHVd-LR1_30       ............................................................   60
CtaHVd-LR1_25       ............................................................   60
                    ****** *  *********************************** *** ****** ***

CtaHVd-LR1_3        AGGTCCGGGAACTTTGTTATGTCAAAAGAGAGGGTAAATCGGGGAAACTCGCCTCCTAGA   120
CtaHVd-LR1_36       ............C...............................................   120
CtaHVd-LR1_22       ............C...............................................   120
CtaHVd-LR1_17       .....................A......................................   120
CtaHVd-LR1_23       ............C...............................................   120
CtaHVd-LR1_20       ............C...............................................   120
CtaHVd-LR1_24       ............C......................................T........   120
CtaHVd-LR1_19       ............C...............................................   120
CtaHVd-LR1_18       ............C...............................................   120
CtaHVd-LR1_21       ............C...............................................   120
CtaHVd-LR1_11       ............C..........................................A....   120
CtaHVd-LR1_5        ............C...............................................   120
CtaHVd-LR1_10       ............C...............................................   120
CtaHVd-LR1_2        ............C.....G.........................................   120
CtaHVd-LR1_33       .....................A......................................   120
CtaHVd-LR1_32       ............C...............................................   120
CtaHVd-LR1_31       ............C...............................................   120
CtaHVd-LR1_14       ............C...............................................   120
CtaHVd-LR1_9        ............C...............................................   120
CtaHVd-LR1_35       ............C...............................................   120
CtaHVd-LR1_12       ............C...............................................   120
CtaHVd-LR1_6        ............C........A......................................   120
CtaHVd-LR1_28       ............C...............................................   120
CtaHVd-LR1_8        ............C......................................T........   120
CtaHVd-LR1_34       ............C...............................................   120
CtaHVd-LR1_29       ............................................................   120
CtaHVd-LR1_27       .......................................T....................   120
CtaHVd-LR1_16       ............C...............................................   120
CtaHVd-LR1_30       ............................................................   120
CtaHVd-LR1_25       ............C...............................................   120
                    ************ ***** ** ***************** *********** *** ****
```

Primer CH1 Rev   Primer CH2 For

```
CtaHVd-LR1_3     AATGAGGGCTCTGCTCTTACAGTATAATGGCGAGGTACCGTGCAGTTAGCAAGGGATTGC   180
CtaHVd-LR1_36    ....................T.......................................   180
CtaHVd-LR1_22    ...........A................................................   180
CtaHVd-LR1_17    ...................A......T.................................   180
CtaHVd-LR1_23    ............................................................   180
CtaHVd-LR1_20    ...................A........................................   180
CtaHVd-LR1_24    ...................A.........A..............................   180
CtaHVd-LR1_19    ...................A........................................   180
CtaHVd-LR1_18    ...................A...............A........................   180
CtaHVd-LR1_21    ...................A........................................   180
CtaHVd-LR1_11    ............................................................   180
CtaHVd-LR1_5     ............................................................   180
CtaHVd-LR1_10    ............................................................   180
CtaHVd-LR1_2     ............................................................   180
CtaHVd-LR1_33    ...................A........................................   180
CtaHVd-LR1_32    ............................................................   180
CtaHVd-LR1_31    ...................A........................................   180
CtaHVd-LR1_14    ............................................................   180
CtaHVd-LR1_9     ............................................................   180
CtaHVd-LR1_35    ............................................................   180
CtaHVd-LR1_12    ............................................................   180
CtaHVd-LR1_6     ............................................................   180
CtaHVd-LR1_28    ............................................................   180
CtaHVd-LR1_8     ............................................................   180
CtaHVd-LR1_34    ............................................................   180
CtaHVd-LR1_29    .........C.........A........................................   180
CtaHVd-LR1_27    ...................A........................................   180
CtaHVd-LR1_16    .........C..................................................   180
CtaHVd-LR1_30    .........C.........A........................................   180
CtaHVd-LR1_25    .........C.........A........................................   180
                 ********* * *******  ***** ** ***** ************************

CtaHVd-LR1_3     TAGCTTCTTTCATTTGGGAAACCAATGAGAGAAGACCTAGTTTACCCGCTTGCTTCGTGC   240
CtaHVd-LR1_36    .........................C..............CCC..........CC..C..   240
CtaHVd-LR1_22    ............................AG..............................   240
CtaHVd-LR1_17    ............................................................   240
CtaHVd-LR1_23    ............................AG..............................   240
CtaHVd-LR1_20    ............................AG..............................   240
CtaHVd-LR1_24    ............................................................   240
CtaHVd-LR1_19    ............................................................   240
CtaHVd-LR1_18    ............................................................   240
CtaHVd-LR1_21    ............................................................   240
CtaHVd-LR1_11    ............................AG..............................   240
CtaHVd-LR1_5     .......C....................AG..............................   240
CtaHVd-LR1_10    ............................AG..............................   240
CtaHVd-LR1_2     ............................AG..............................   240
CtaHVd-LR1_33    ............................................................   240
CtaHVd-LR1_32    ............................AG..............................   240
CtaHVd-LR1_31    ............................AG..............................   240
CtaHVd-LR1_14    ............................AG..............................   240
CtaHVd-LR1_9     ............................AG..............................   240
CtaHVd-LR1_35    ............................AG................T.............   240
CtaHVd-LR1_12    ............................AG................T.............   240
CtaHVd-LR1_6     ............................AG................T.............   240
CtaHVd-LR1_28    ............................AG..............................   240
CtaHVd-LR1_8     ............................AG..............................   240
CtaHVd-LR1_34    ............................AG..............................   240
CtaHVd-LR1_29    ............................................................   240
CtaHVd-LR1_27    ............................................................   240
CtaHVd-LR1_16    ............................................................   240
CtaHVd-LR1_30    ..............................................T.............   240
CtaHVd-LR1_25    ............................................................   240
                 ******* ***************** **  **********   *** ******  ** **
```

```
CtaHVd-LR1_3        GGGAACCCGTTCACTTAACCGAAAGCGCAAGCTTCTCAGGTTTTGGAAGTGAGAGTCCTG    300
CtaHVd-LR1_36       ..............CC................CC.......C.......A...T......    300
CtaHVd-LR1_22       .................................................A...T......    300
CtaHVd-LR1_17       ............................................................    300
CtaHVd-LR1_23       ............................................................    300
CtaHVd-LR1_20       .................................................A...T......    300
CtaHVd-LR1_24       ............................................................    300
CtaHVd-LR1_19       ............................................................    300
CtaHVd-LR1_18       ............................................................    300
CtaHVd-LR1_21       ............................................................
CtaHVd-LR1_11       ......................G..........................A...T......    300
CtaHVd-LR1_5        ............................................................    300
CtaHVd-LR1_10       .................................................A...T......    300
CtaHVd-LR1_2        ............................................................    300
CtaHVd-LR1_33       ............................................................    300
CtaHVd-LR1_32       .................................................A...T......    300
CtaHVd-LR1_31       ............................................................    300
CtaHVd-LR1_14       .................................................A..........    300
CtaHVd-LR1_9        .................................................A...T......    300
CtaHVd-LR1_35       .................................................A...T......    300
CtaHVd-LR1_12       .................................................A...T......    300
CtaHVd-LR1_6        .................................................A...T......    300
CtaHVd-LR1_28       .................................................A...T......    300
CtaHVd-LR1_8        .................................................A...T......    300
CtaHVd-LR1_34       .................................................A...T......    300
CtaHVd-LR1_29       ............................................................    300
CtaHVd-LR1_27       ............................................................    300
CtaHVd-LR1_16       ..............CC............................................    300
CtaHVd-LR1_30       ............................................................    300
CtaHVd-LR1_25       ............................................................    300
                    **************  ****** *********  *** *** ******* *** ******

CtaHVd-LR1_3        ATAATGCCTCTGAATTGAA-AAACAAAACTCGGCTCCTCAAAAGCAATGTGGATTTCGAC    359
CtaHVd-LR1_36       ....CC.............A......G.......C.........................    360
CtaHVd-LR1_22       .....A..............G.....G.................................    359
CtaHVd-LR1_17       ....CA...........T..........................................    359
CtaHVd-LR1_23       ....CA....................G.................................    359
CtaHVd-LR1_20       ....CA..............G.....G.................................    359
CtaHVd-LR1_24       ....C...................T.G.................................    359
CtaHVd-LR1_19       ....CA....................GT................................    359
CtaHVd-LR1_18       ....CA..............G.....G.................................    359
CtaHVd-LR1_21       ....CA....................G....A............................    359
CtaHVd-LR1_11       ....CA...........T.TG.....G.................................    360
CtaHVd-LR1_5        ....CA....................G.................................    359
CtaHVd-LR1_10       ....CA....................G.................................    359
CtaHVd-LR1_2        ....CA...........T..........................................    359
CtaHVd-LR1_33       ....CA....................G.................................    359
CtaHVd-LR1_32       ....CA..............G.....G.................................    359
CtaHVd-LR1_31       ....CA....................GT................................    359
CtaCtaHVd-LR1_14    ....CA....................G.................................    359
CtaHVd-LR1_9        ....CA....................G.................................    359
CtaHVd-LR1_35       ....CA....................G.....................A...........    359
CtaHVd-LR1_12       ....CA............G.......G.................................    359
CtaHVd-LR1_6        ....CA....................G.................................    359
CtaHVd-LR1_28       ....CA....................G.................................    359
CtaHVd-LR1_8        ....CA....................G.................................    359
CtaCtaHVd-LR1_34       ....CA....................G.................................    359
CtaHVd-LR1_29       ....CA................T.....................................    359
CtaHVd-LR1_27       ....CA....................G.................................    359
CtaHVd-LR1_16       ....CA....................G.................................    359
CtaHVd-LR1_30       ..........................G.................................    359
CtaHVd-LR1_25       ....CA....................G.................................    359
                    ****  ***********    * * *  *** ** ************* ***********
```

```
CtaHVd-LR1_3    CTGCTAAAAGTCTCATCAGGGCAGCCAGCGACAGGCGCCTATCCACAACCACTCGTTCTT    419
CtaHVd-LR1_36   ....C.......................................................    420
CtaHVd-LR1_22   .....TTT.........G....G.....................................    419
CtaHVd-LR1_17   .............................A..............................    419
CtaHVd-LR1_23   ............................................................    419
CtaHVd-LR1_20   ............................................................    419
CtaHVd-LR1_24   ............................................................    419
CtaHVd-LR1_19   ....C.......................................................    419
CtaHVd-LR1_18   ............................................................    419
CtaHVd-LR1_21   ............................................................    419
CtaHVd-LR1_11   .......T....................................................    420
CtaHVd-LR1_5    ............................................................    419
CtaHVd-LR1_10   ............................................................    419
CtaHVd-LR1_2    ............................................................    419
CtaHVd-LR1_33   .........A..................................................    419
CtaHVd-LR1_32   .................G..........................................    419
CtaHVd-LR1_31   ............................................................    419
CtaHVd-LR1_14   ............................................................    419
CtaHVd-LR1_9    ............................................................    419
CtaHVd-LR1_35   .............................A..............................    419
CtaHVd-LR1_12   ............................................................    419
CtaHVd-LR1_6    ............................................................    419
CtaHVd-LR1_28   ............................................................    419
CtaHVd-LR1_8    ............................................................    419
CtaHVd-LR1_34   ............................................................    419
CtaHVd-LR1_29   .............................A..............................    419
CtaHVd-LR1_27   .................G..........................................    419
CtaHVd-LR1_16   ....C.......................................................    419
CtaHVd-LR1_30   ..............G.............................................    419
CtaHVd-LR1_25   .................G..........................................    419
                ****    * **** ** **** ****** ******************************

                          Primer CH5 Rev      Primer CH6 For

CtaHVd-LR1_3    CTTCAGAAGTTGCGGAGGTCGGTTTG-GGATCCAACCTAGCGGTTAAATCAAATTTT--A    476
CtaHVd-LR1_36   .........CC...........CC.............C.....CC...............    477
CtaHVd-LR1_22   ..........................G.................................    477
CtaHVd-LR1_17   ..........................G.......................G.........    477
CtaHVd-LR1_23   ..........................G.................................    477
CtaHVd-LR1_20   ..........................G.................................    477
CtaHVd-LR1_24   ..........................G.................................    477
CtaHVd-LR1_19   ..........................G.................................    477
CtaHVd-LR1_18   ..........................G.................................    477
CtaHVd-LR1_21   ..........................G.................................    477
CtaHVd-LR1_11   ..........C.....................T...........................    477
CtaHVd-LR1_5    ................................T........................TA.    478
CtaHVd-LR1_10   ..........C.....................TG...........GG.............    476
CtaHVd-LR1_2    ..........C.....................T............GG.............    476
CtaHVd-LR1_33   ................................T...........................    476
CtaHVd-LR1_32   ..........C.....................T.G..........G..............    476
CtaHVd-LR1_31   ................................T...........................    476
CtaHVd-LR1_14   ............................................................    476
CtaHVd-LR1_9    ................................T............G..............    476
CtaHVd-LR1_35   ................................T...........................    476
CtaHVd-LR1_12   ..........C.....................T...........................    476
CtaHVd-LR1_6    ..........C.....................T...........................    476
CtaHVd-LR1_28   ..........C.....................T...........................    476
CtaHVd-LR1_8    ..........C.....................T...........................    476
CtaHVd-LR1_34   ..........C.....................T...........................    476
CtaHVd-LR1_29   ....................................T.......................    476
CtaHVd-LR1_27   ..........C.....................T............GG.............    476
CtaHVd-LR1_16   ............................................................    476
CtaHVd-LR1_30   ..........C.....................T...........................    476
CtaHVd-LR1_25   ..........C.....................T...........................    476
                *********  ***********  ** *****      *****    *** ******  *
```

```
CtaHVd-LR1_3        CTTTGTTAAACCTGCTTAGTTCGGACCTGACACCAGCCCCGTCGCTAATGTTGAAGGAAG      536
CtaHVd-LR1_36       .....CC.....A..CC..CC.A..................C..................      537
CtaHVd-LR1_22       ............A.........A.....................................      537
CtaHVd-LR1_17       ............................................................      537
CtaHVd-LR1_23       ......A...............A.....................................      537
CtaHVd-LR1_20       ......A..G..................................................      537
CtaHVd-LR1_24       ......A.....................................................      537
CtaHVd-LR1_19       ............................................................      537
CtaHVd-LR1_18       ......A...............A.....................................      537
CtaHVd-LR1_21       ......A.....................................................      537
CtaHVd-LR1_11       ......A...............A.....................................      537
CtaHVd-LR1_5        T.....A...............A.....................................      538
CtaHVd-LR1_10       ........G...A....G....A........G............................      536
CtaHVd-LR1_2        ............A.........A.....................................      536
CtaHVd-LR1_33       ......A...............A.....................................      536
CtaHVd-LR1_32       ........G...A.........A.....................................      536
CtaHVd-LR1_31       ......A...............A.....................................      536
CtaHVd-LR1_14       ............A.........A.....................................      536
CtaHVd-LR1_9        ......A.....G.........A.....................................      536
CtaHVd-LR1_35       ......A.....G.........A.....................................      536
CtaHVd-LR1_12       ............A.........A.....................................      536
CtaHVd-LR1_6        ......A.....G.........A.....................................      536
CtaHVd-LR1_28       ......A.G.............A.....................................      536
CtaHVd-LR1_8        ......................A.....................................      536
CtaHVd-LR1_34       ......................A.....................................      536
CtaHVd-LR1_29       ............G.........A.....................................      536
CtaHVd-LR1_27       .......GG........G....A.....................................      536
CtaHVd-LR1_16       ......A........C............................................      536
CtaHVd-LR1_30       ............G.........A.....................................      536
CtaHVd-LR1_25       ............................................................      536
                     ****     ** **   *  * ******** ********* ******************


CtaHVd-LR1_3        AAGTTGTGGGTGTA 550
CtaHVd-LR1_36       .............. 551
CtaHVd-LR1_22       .............. 551
CtaHVd-LR1_17       .............. 551
CtaHVd-LR1_23       .............. 551
CtaHVd-LR1_20       .............. 551
CtaHVd-LR1_24       .............. 551
CtaHVd-LR1_19       .............. 551
CtaHVd-LR1_18       .............. 551
CtaHVd-LR1_21       .............. 551
CtaHVd-LR1_11       .............. 551
CtaHVd-LR1_5        .............. 552
CtaHVd-LR1_10       .............. 550
CtaHVd-LR1_2        .............. 550
CtaHVd-LR1_33       .............. 550
CtaHVd-LR1_32       .............. 550
CtaHVd-LR1_31       .............. 550
CtaHVd-LR1_14       .............. 550
CtaHVd-LR1_9        .............. 550
CtaHVd-LR1_35       .............. 550
CtaHVd-LR1_12       .............. 550
CtaHVd-LR1_6        .............. 550
CtaHVd-LR1_28       .............. 550
CtaHVd-LR1_8        .............. 550
CtaHVd-LR1_34       .............. 550
CtaHVd-LR1_29       .............. 550
CtaHVd-LR1_27       .............. 550
CtaHVd-LR1_16       .............. 550
CtaHVd-LR1_30       .............. 550
CtaHVd-LR1_25       .............. 550
                    **************
```

Figure S4. Multiple-sequence alignment of full-length cDNAs of citrus transiently associated hammerhead viroid-like RNA 1 variants amplified with primer pairs CH1 Rev/CH2 For (from variant CtaHVd-LR1_2 to

CtaHVd-LR1_12), CH3 Rev/CH4 For (variants CtaHVd-LR1_14 to CtaHVd-LR1_24) and CH5 Rev/CH6 For (variants CtaHVd-LR1_25 to CtaHVd-LR1_36). Names of the sequence variants and nucleotide positions in the alignment are reported on the left and the right, respectively. Regions covered by reverse (Rev) and forward (For) primers are marked by arrows (blue and green, respectively). Nucleotide identity and gaps with respect to the reference variant are indicated by dots and dashes, respectively. Regions involved in the formation of (+) and (-) hammerhead structures are in red and blue, respectively, with the nucleotides conserved in most natural hammerhead structures on a grey background. The reference variant (CtaHVd-LR_3) reported at the top shares the highest identity with the consensus sequence of the alignment, from which it differs in six nucleotides not located in the conserved HHRzs motifs.

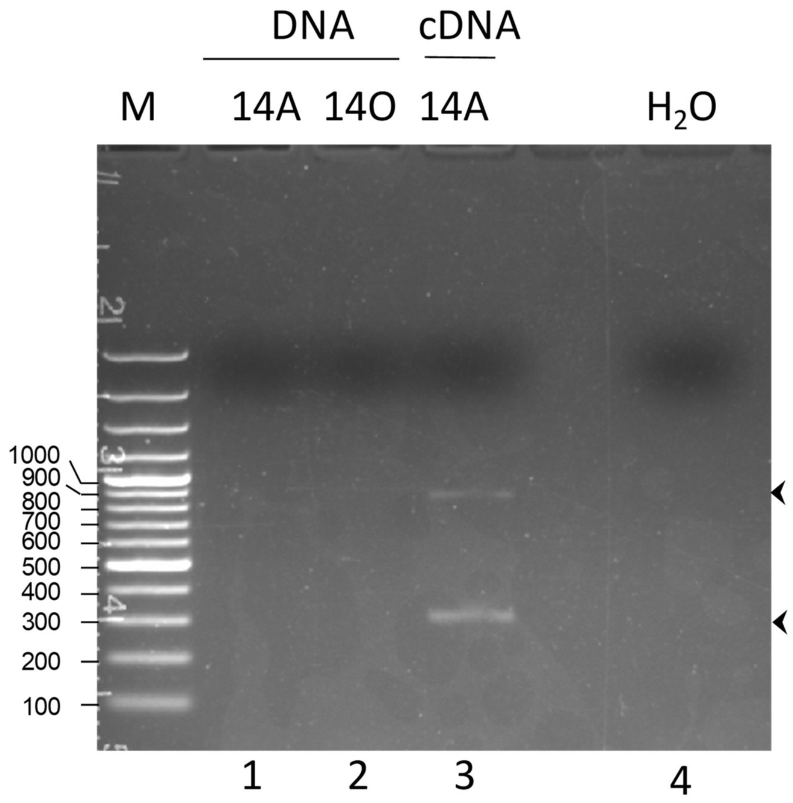

Figure S5. PCR and RT-PCR assays using DNA (lane 1) and RNA (lane 3) preparations from isolate 14A, respectively. A DNA preparation from the citrus isolate 14O (lane 2) and a mix in which water replaced DNA (lane 4) were used as negative controls for the PCR assay. Molecular Marker (M) is 100 bp ladder (New England biolabs). Arrowhead on the left indicate the HVd-LR1 amplicon of 304 nt expected to be amplified by RT-PCR from isolate 14A and a larger amplicon deriving from the amplification of a larger than unit CtaHVd-LR1 cDNA.
